# Anesthesiology Control Tower: Feasibility Assessment to Support Translation (ACT-FAST)—a feasibility study protocol

**DOI:** 10.1186/s40814-018-0233-4

**Published:** 2018-01-25

**Authors:** Teresa M. Murray-Torres, Frances Wallace, Mara Bollini, Michael S. Avidan, Mary C. Politi

**Affiliations:** 1 Department of Anesthesiology, Washington University School of Medicine, 660 Euclid Ave, Camps Box 8054, St. Louis, MO 63110 USA; 2 Washington University School of Medicine, 660 Euclid Ave, St. Louis, MO 63110 USA; 3 Department of Surgery, Washington University School of Medicine, 600 Euclid Ave, St. Louis, MO 63110 USA; 4 Washington University School of Public Health, 600 Euclid Ave, St. Louis, MO 63110 USA

**Keywords:** Usability, Feasibility, Telemedicine, Clinician decision support, Health information technology, Human-computer interaction

## Abstract

**Background:**

Major postoperative morbidity and mortality remain common despite efforts to improve patient outcomes. Health information technologies have the potential to actualize advances in perioperative patient care, but failure to evaluate the usability of these technologies may hinder their implementation and acceptance. This protocol describes the usability testing of an innovative telemedicine-based intra-operative clinical support system, the Anesthesiology Control Tower, in which a team led by an attending anesthesiologist will use a combination of established and novel information technologies to provide evidence-based support to their colleagues in the operating room.

**Methods:**

Two phases of mixed-methods usability testing will be conducted in an iterative manner and will evaluate both the individual components of the Anesthesiology Control Tower and their integration as a whole. Phase I testing will employ two separate “think-aloud” protocol analyses with the two groups of end users. Segments will be coded and analyzed for usability issues. Phase II will involve a qualitative and quantitative in situ usability and feasibility analysis. Results from each phase will inform the revision and improvement of the Control Tower prototype throughout our testing and analysis process. The final prototype will be evaluated in the form of a pragmatic randomized controlled clinical trial.

**Discussion:**

The Anesthesiology Control Tower has the potential to revolutionize the standard of care for perioperative medicine. Through the thorough and iterative usability testing process described in this protocol, we will maximize the usefulness of this novel technology for our clinicians, thus improving our ability to implement this innovation into the model of care for perioperative medicine.

**Trial registration:**

The study that this protocol describes has been registered in clinicaltrials.gov as NCT02830126.

## Background

Despite major advancements in the safety of anesthetic techniques and therapeutics, the risk of patient morbidity and mortality related to surgery persists. Some of this risk is unavoidable and either is inherent to the nature of the surgical procedure itself or is attributable to a characteristic of the patient that is not in an immediate way modifiable [1–3]. There are, however, factors that do fall under the control of the anesthetic team that have been shown to affect the patient’s immediate and long-term health [4–7]. Information technology-driven decision support has been shown to optimize management of these factors, leading to improvement in physiological measures, such as blood pressure stability [8] and glucose control [9, 10]. This decision support can aid members of the anesthesia care team, who often experience information overload in the operating room that can limit their implementation of evidence-based practice. Health IT tools that work to advance and implement decision support systems are being championed in the demand for enhanced quality in health care [11, 12]. A critical component of the implementation process is the inclusion of usability analysis of the health IT throughout its lifecycle, beginning in the development phase and continuing into the post-implementation period [13]. Usability is an essential feature of the successful implementation of novel information technologies [14–16] and has significant impact on productivity and performance [13] in addition to the acceptance and safety of health IT systems [13].

At our institution, we are developing a novel telemedicine-based intervention for the operating room, the Anesthesiology Control Tower (ACT), which is similar in concept to an air traffic control tower for a busy airport. The clinicians in the Anesthesiology Control Tower (ACTors) will monitor active operating rooms (ORs) in real time by using several electronic health records at our institution. Just as an air traffic control tower monitors each aircraft and delivers additional information and alerts to the pilot and co-pilot, the ACT will engage with teams of anesthesia clinicians in a similar fashion to assist them in providing safe, effective, and efficient care for their patients. The ACT intervention will be evaluated in the form of a pragmatic, comparative effectiveness randomized controlled trial (NCT02830126). However, prior to the implementation of the ACT, it will be crucial to ensure that it is a useful and usable resource for the clinicians for whom it is developed, as usability is a critical feature of the successful implementation of novel information technologies [14–16], with a significant impact on productivity and performance [13] in addition to the acceptance and safety of health IT systems [13]. The primary objective of the protocol described in this paper is to evaluate the usability of the ACT by gathering perceptions of key stakeholders and end users of the ACT and secondarily to assess the feasibility of its implementation in routine care, prior to conducting a pragmatic trial evaluating the ACT. Usability testing will be performed in an iterative manner throughout the design and implementation stages and will focus on outcomes related to effectiveness, efficiency, and acceptability. This protocol describes two phases of mixed-methods (qualitative and quantitative) usability and feasibility testing [15, 17–20] of the ACT structure, including the software platforms that it employs.

## Methods

**Fig. 1:**
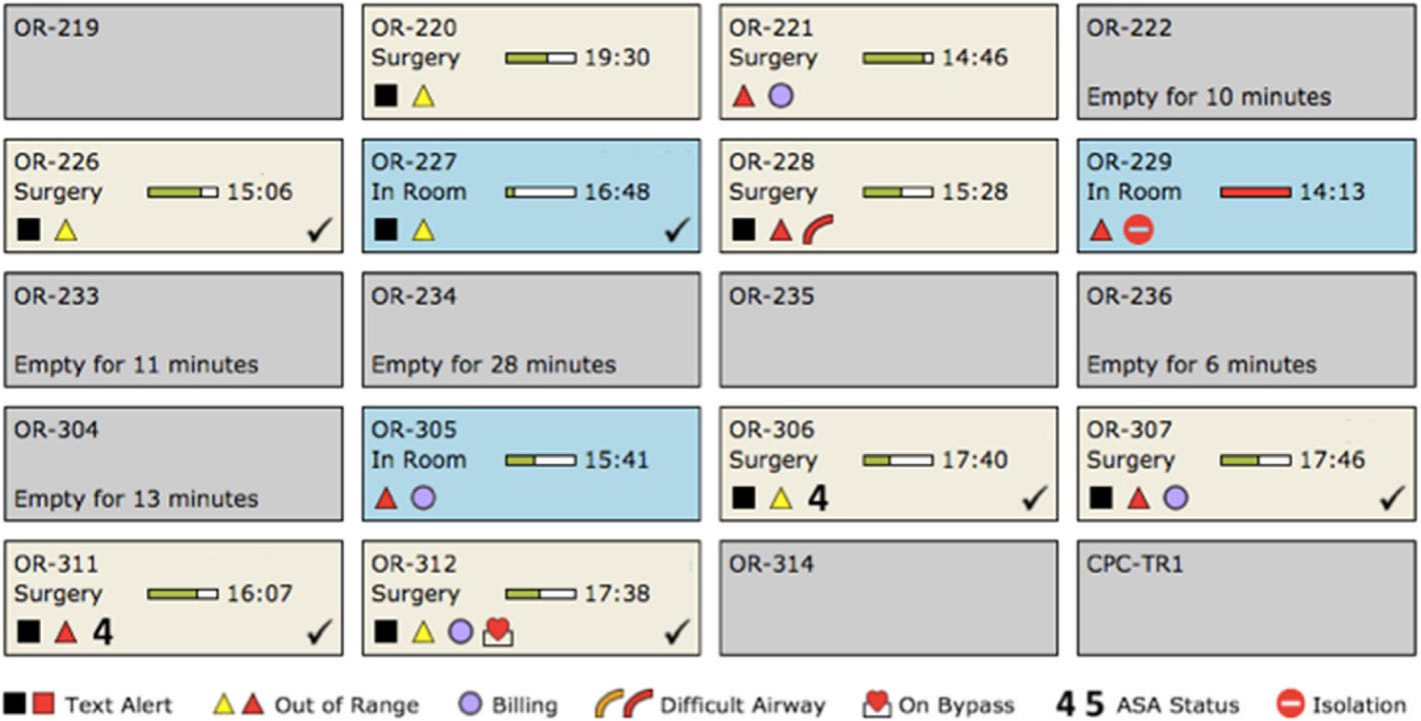
AlertWatch Tower Mode census view. From this view, clinicians in the ACT can obtain a brief overview of all the patients in the ORs. Alerts and abnormal physiologic and laboratory parameters are represented by squares and triangles, respectively; checkmarks indicate alerts that must be addressed by the ACT. These groups of alerts are unique to the AW Tower Mode and will be refined based on the results of the present study. Clicking on an OR accesses the detailed information for that OR

**Fig. 2:**
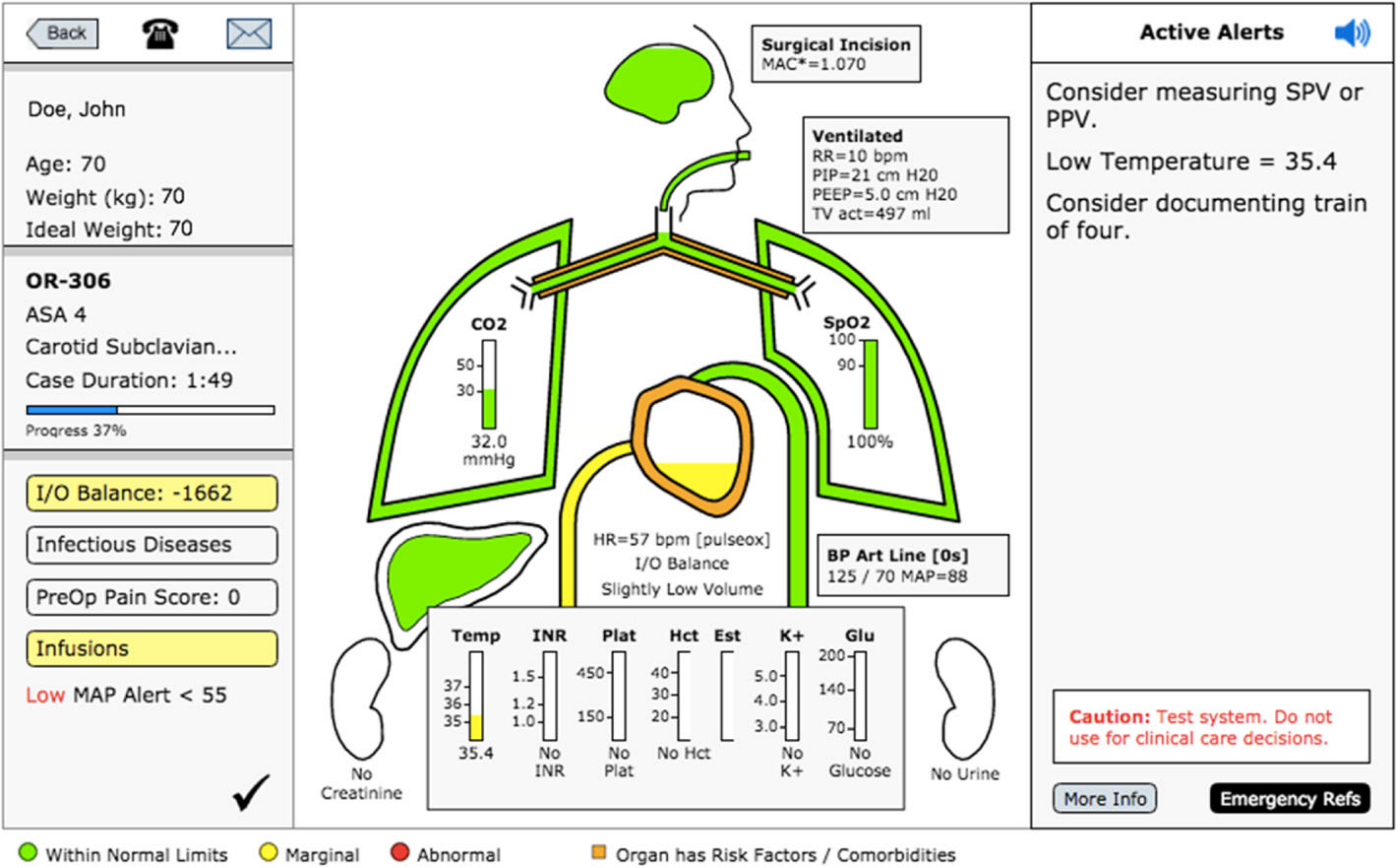
AlertWatch Tower Mode patient display. Organ systems are depicted and labeled with relevant physiologic variables and values. Colors outlining organs indicate normal (green), marginal (yellow), or abnormal function (red). The left side of the display shows patient characteristics and case information. Information regarding the actual patient’s comorbidities can be accessed by selecting the organ system or laboratory study of interest. Text alerts are present on the right-hand side of the screen. The black checkmark at the bottom of the left panel indicates that there is an active alert for the ACT clinicians to address; clicking on the checkmark opens the case review dialogue (Figure 3)

**Fig. 3:**
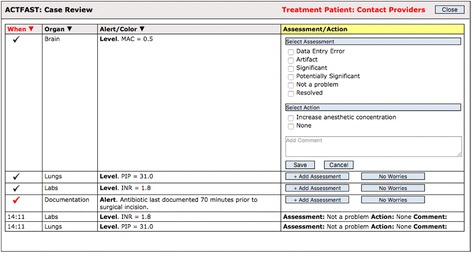
Case review. This popup window allows physicians in the ACT (ACTors) to document their assessment of alerts and what actions they would recommend. This is a feature of AlertWatch that is unique to the ACT Tower Mode platform. ACTors successfully assess and address an alert by documenting their assessment of the significance of the alert and by documenting what action they would recommend taking, if any

The full description of ACT is available in Appendix 1. This includes a description of one of the software programs used in the ACT, called AlertWatch® (Ann Arbor, MI), which is a clinical monitoring and alerting system (Appendix 1, Figs. 1, 2 and 3). The specific and unique platform described in this protocol, AlertWatch (AW) Tower Mode, will be customized and refined through this research. While the developers of AlertWatch will be involved in refinements of the ACT based on the findings of our study, they will have no input on the design of the study nor how it is carried out and no involvement in the data collection and analysis process. The initial design of the AW Tower Mode is based on input from experts in clinical anesthesiology and medical informatics. We anticipate that prototype revision and improvement will occur throughout the usability testing and analysis process.

Two phases of usability testing will be conducted in series, as described fully below. A mixed-methods approach of qualitative and quantitative analysis will be employed. The first phase of testing will be an exploratory analysis that employs a “think-aloud” approach, which is well suited for identifying key usability issues and barriers to adoption of new technologies [21]. It will include parallel testing with two groups of end users (Table 1). The second phase of testing will involve in situ use of the ACT by end users and will further focus on the outcomes of efficiency, efficacy, and learnability of and satisfaction with the ACT [22, 23].

**Table 1:** Participant groups at each stage of testing

Stage of testing	Description	Potential Participant Groups
Phase Ia	“Think-aloud” analysis for physicians staffing ACT	Attending anesthesiologists
		Anesthesiology residents
Phase Ib	“Think-aloud” analysis for clinicians receiving ACT support	Attending anesthesiologists
		Anesthesiology residents
		Certified registered nurse anesthetists
Phase II	Mixed in situ usability and feasibility analysis	Attending anesthesiologists
		Anesthesiology residents

Participants in this study will be recruited from the department of anesthesiology through approved use of the departmental distribution list, which is the most common method of communication within the department. An initial recruitment email will be sent to all the members of each relevant participant group (Table 1). Additional study information will be provided to any interested parties, and those who express interest in joining the study will be formally consented for participation.

During the study, access to data files will be strictly limited to members of the research team. All computer files will be stored on an encrypted server, with additional password protection for any file that contains identifying information. Physical copies of surveys etc. will be stored in a locked location to which only the research team has access. All physical data files and all recordings will be destroyed at a pre-specified time after collection.

### Phase I: usability analysis with a “think-aloud” protocol

#### Phase Ia

Participants will be recruited via email from the groups of clinicians who would potentially staff the ACT (ACTors), namely, attending anesthesiologists and senior anesthesiology residents. All attending and senior resident anesthesiologists in the department will be eligible to participate. Eight to ten participants are an appropriate number to identify the majority of surface-level usability problems [24], and three to five participants per testing round are cost-efficient, maximizing identification of issues while reducing redundancy [25]. A total of 12–15 participants will participate.

#### Phase Ib

Participants will be recruited via email from the groups of clinicians who are potential recipients of the ACT support (clinicians in the OR—or CORs), namely, attending anesthesiologists, anesthesiology residents, and certified registered nurse anesthetists (CRNAs). A total of 10–15 participants will be included. There will be no overlap with participants in phase Ia, but there are no other exclusion criteria.

#### Procedure

##### Phase Ia: “think-aloud” with ACTors

Participants will receive an orientation to the ACT and a description of the tasks that they are to accomplish. Sessions will be conducted by the authors in the room that houses the ACT (TM, FW, MB); one or two authors will be present per session without additional observers. These authors are members of the department of anesthesiology who do not have any supervisory roles in relation to participants in the study (resident physician, medical student, and research staff member, respectively). These authors will receive training on the administration of usability testing scenarios. During the sessions, participants will use available clinical applications to monitor active operating rooms and address alerts generated by the AW Tower Mode platform. Alerts are addressed by documenting an assessment and recommended action for each alert. Users will verbalize all thoughts, feelings, and questions as they navigate through specific aspects of the software programs and the ACT layout and design to complete their tasks. The research team members will prompt participants only if 20 s [21] or more elapses without verbalization from the participant. Sessions will be audio recorded. Observations and field notes from the research team will be included to provide additional insight. Participants will complete 20 min of testing. This will be followed by a debriefing session that will employ open-ended questions to obtain feedback on specific issues (Appendix 2). The total length of sessions will be approximately 50–60 min.

At the end of each session, participants will complete the NASA Task Loading Index (NASA-TLX [26–28]), the 10-item System Usability Scale (SUS [29]), and the 19-item Computer System Usability Questionnaire (CSUQ [30]). The SUS and the CSUQ are appropriate for subjective ratings of usability. The SUS offers a final score that ranges from 0 to 100, with a “passable” score above 70 [31]. The CSUQ offers a total score ranging from 0 to 7, in addition to three subscale scores (system usefulness, information quality, and interface quality).

##### Phase Ib: “think-aloud” with CORs

Sessions will take place in a conference room on the medical campus of the hospital complex and will be administered by one author (TM) with a second author as an observer (FW). Participants will be informed of the purpose and format of the procedure and will receive an orientation to how the ACT will function. After this, open-ended questions will be used to prompt participants to provide their initial reactions to the ACT intervention. Subsequently, the research team member will present clinical scenarios that invite the participant to think about interacting with the ACT as a recipient of the ACT support. Participants will be instructed to imagine themselves in each scenario and to voice all thoughts that arise as they do so. After participants have had the opportunity to share their thoughts freely for each scenario, a short series of open-ended questions (Appendix 2) will be used to obtain COR feedback with regard to specific features of the ACT, such as the usefulness of particular alerts and of different methods of communication with the ACT. Sessions will be audio recorded. Field notes and observations from the research team member will again be included to provide additional insight. Sessions are expected to last approximately 30 min.

#### Data analysis

Given that the “think-aloud” and similar methods often slow task completion and thought processes [21], the focus of analysis will be on qualitative usability data via content analysis, although a few relevant quantitative measures will be included. Participant demographics will be reported with descriptive statistics. Mean scores from the SUS, the NASA-TXL, and the overall CSUQ score and subscale scores will be calculated and reported in addition to 95% confidence intervals. Quantitative data from phase Ia will include time to task completion and rate of task completion.

Audio recordings from each participant in both phase Ia and phase Ib will be professionally transcribed. The investigators will review the transcripts to identify themes and create a codebook with themes and subthemes surrounding the usability of the ACT [32]. We will use a qualitative data analysis software program (NVivo®) to organize and code the transcripts. We will perform a content analysis of key themes based on the frequency and level of emotive force expressed by participants. Themes will be examined within and across user groups (physician anesthesiologists, CRNAs). Research team members trained in qualitative methods will perform the coding independently until inter-rater reliability is reached. Minor discrepancies will be resolved through discussion and consensus. If there are major discrepancies, we will refine the codebook and recode.

Decisions to modify components of the pilot ACT during the usability testing will be determined by the investigative team, based on participant feedback, and will occur in an iterative fashion.

### Phase II: in situ usability and feasibility testing

#### Participants

Participants will include groups of clinicians who would be eligible to staff the ACT (ACTors), namely attending anesthesiologists and anesthesiology residents, who will be recruited via email. Over the course of this phase, we expect to include 10 subjects [24, 25]. Subjects who complete phase Ia or phase Ib will be eligible to participate in phase II. Resident physicians will participate for ten consecutive business days, due to scheduling constraints at our institution. Attending anesthesiologists will participate one business day at a time and may participate more than once.

#### Procedure

During the sessions, ACTors will use several different software programs to remotely monitor active operating room locations in real time. No research staff will be present. Orientation videos and documents will be provided to participants. They will address alerts that are created within AW Tower Mode (see Appendix 1), but they will not interact with CORs. Other than the lack of interaction between ACTors and CORs, this testing involves a functional prototype of AW Tower Mode and live software programs. A log of all alerts and ACTor responses, including ACTor comments on individual alerts and the documented level of significance for each alert, will be automatically generated and stored on a secure server.

All participants will complete the aforementioned 10-item System Usability Scale (SUS [29]), the 19-item Computer System Usability Questionnaire (CSUQ [30]), and the NASA Task Load Index (NASA-TLX [26]). Resident physicians will complete these scales on days 1, 5, and 10. Attending physicians will complete these scales each day that they are in the ACT. In addition, ACTors will have the opportunity to provide written feedback on a daily basis.

#### Data analysis

This phase of in situ testing will allow us to further evaluate the usability of the ACT and the feasibility of implementation. Demographics will be included with descriptive statistics. Mean scores and 95% confidence intervals for the SUS, the NASA-TXL, and the CSUQ scale and subscale will be calculated and reported for assessment of subjective satisfaction and usability. Any written feedback from participants will be coded using standard techniques for analyzing qualitative data [33]. The feasibility of implementing the ACT will also be examined through analysis of AW data logs for measures of effectiveness and efficiency in this near real-world setting [15, 17, 19, 34–36]. Effectiveness, or the degree to which users are able to succeed in achieving their goals [20], can be described as the “accuracy and completeness” with which tasks are accomplished [34]. Methods of assessing effectiveness include evaluating the quantity and quality of task completion [17, 34]. In this phase of in situ testing, we will assess effectiveness by examining data such as the number of alerts that are successfully addressed and the number of patients that are evaluated. We will also evaluate some measures of efficiency such as the rate at which alerts are addressed, rate of missed alerts, and number of errors.

Patterns in alert responses will also be analyzed. Learnability as part of a feasibility assessment will be determined by evaluating changes in performance of ACTors across levels of experience. The usefulness of individual alerts will be determined by the frequency with which they are rated as significant or insignificant. Changes to the ACT based on information obtained throughout this phase, including the revision of AW alerts, will be made based on the judgment of the investigative team.

## Discussion

Many barriers exist to the implementation of novel health information technologies. Thorough and continual evaluation of the usability of such technologies is critical. A variety of approaches to usability assessment have been employed. These include cognitive walk-through methods [37], focus groups [16], surveys [38], and “think-aloud” protocol analysis [37, 39–42]. A combination of methods is often more powerful than single methods in isolation [43]. Furthermore, “near-live” testing often discover concerns that would have otherwise been missed and is important in the evaluation of the feasibility of implementing a new technology [39, 44]. Therefore, in this protocol, we employ a combination of formal and real-world mixed-methods usability testing.

The formal think-aloud usability sessions with two groups of end users in phase I will provide rich information on surface-level usability problems actually encountered by these users [37]. This data will inform the development and refinement of the ACT intervention, particularly in the early phases of design and implementation. “Think-aloud” testing is well suited to identify barriers to adoption for new technologies [12], although limitations of this method include the potential for hindering cognitive processes, particularly for tasks that involve a high cognitive load [45]. We anticipate that the phase II in situ qualitative and quantitative usability testing, with its heightened fidelity, will provide additional and complementary insight into usability and workflow concerns in a more realistic setting. Real-world testing such as this often discovers concerns that would have otherwise been missed in exploratory or formal usability testing [39, 44]. By analyzing a combination of data logs and user questionnaires, we will obtain results pertaining to major usability elements, namely, efficacy, efficiency, learnability, and satisfaction [20, 22, 35, 43, 46], that will allow us to ensure a useful and usable resource for our clinicians.

Usability testing throughout the lifecycle of a given technology is a crucial component of the successful implementation of such technologies [36, 43], and the results of our iterative analyses will inform and refine the development of our intervention, particularly the AW Tower Mode platform. This Tower Mode platform is a customized product being specifically created and designed for the purpose of instituting the ACT and for our future randomized controlled trial. Although we are testing with a limited sample size, we anticipate that the results of the usability analysis described in this paper will provide a sufficient breadth and depth of information to allow us to ensure that the platform, which plays an integral role in the ACT, incorporates the actual needs of our users [24].

The planned pragmatic RCT will continue to evaluate user experience in real-world implementation settings to capture additional information about usability. For example, the current study is designed to evaluate the usability of the ACT and its software programs, prior to the initiation of the randomized controlled trial in which ACTors will be providing support to anesthesia clinicians in the OR. The usability of the modes of interaction between ACTors and CORs will not assessed with the present methods, and we do expect this to have an impact on the fully functional ACT. Therefore, in our future randomized controlled trial involving the ACT intervention, we plan to implement post-implementation analyses [13] that evaluate and refine the ACT to ensure that it is a useful and usable resource for our clinicians in an actual real-world setting. We anticipate that some of these analyses will be in the form of surveys with both closed and open-ended responses that are administered to the members of the anesthesia department.

The ACTors in our study will be monitoring up to 50 patients at a time, a novel model for anesthesia care that deviates significantly from the traditional model in which one anesthesiologist is responsible for no more than four rooms. The ACT will demand a high level of cognitive functioning. This level of cognitive functioning necessitates resources that have been thoroughly examined for design and usability flaws. Thus, this series of iterative usability testing and improvement is vital to the development and implementation of our innovative intervention.

## Appendix 1

### ACT description

#### ACTFAST context

The current study describes one part of our larger effort to implement the Anesthesiology Control Tower (ACT), a novel, telemedicine-based intervention for the operating suite. The purpose of the research described in the current protocol is to use iterative usability testing over the course of approximately 6 months, in order to create a maximally useful and usable resource for our clinicians. A second, concurrent study by our group (Anesthesiology Control Tower: Forecasting Algorithms to Support Treatments, or ACTFAST2) is working to develop, refine, and validate forecasting algorithms to predict negative patient trajectories; such algorithms will serve to help inform ACT interventions. Our final phase of investigation will be a yearlong comparative effectiveness randomized controlled trial (Anesthesiology Control Tower: Feedback Alerts to Supplement Treatments, or ACTFAST3; NCT02830126) to demonstrate the utility of the ACT in improving adherence to clinically relevant practice guidelines and to evaluate the effect of the ACT on surrogate measures of patient outcome. In this trial, each of our institution’s adult ORs will be randomized on a daily basis either to a control group or to an experimental group in which anesthesia providers in those ORs will receive the support of the ACT. This support will be communicated in the form of additional alerts sent by the physicians in the ACT, whose responsibility is to filter and prioritize information, thus maximizing alert quality and minimizing false alarms.

#### ACT technology

One of the key components of the ACT technological infrastructure is an advanced clinical monitoring and display system called AlertWatch ® (Ann Arbor, MI). AlertWatch (AW) uses a multi-threaded Java-based server to collect and integrate clinical information from multiple distinct data sources for presentation in a simplified and intuitive manner (see Figs. 1, 2 and 3). AW provides alerts based on its continual analysis of the current patient state; when patient parameters fall outside of predetermined set points, AW triggers written and auditory alarms within the patient display and on the census view. The AW Tower Mode platform used in this study is distinct from the commercially available product in many ways and will be refined based on the results of the present study.

AlertWatch and all additional electronic records used by our hospital are available via secure Internet connection to clinicians at any physical location in the world, allowing for the creation of our remote ACT. This includes our anesthesia information management system, MetaVision (iMDsoft®, Needham, MA; Centricity, General Electric Healthcare), which contains both manually entered data and automatically recorded physiologic data for the perioperative period.

#### ACT structure

The ACT is physically housed within the hospital complex and is remoted from the physical operating rooms. This room includes three computers: two with two monitors and one with three monitors (one being a flat panel TV screen). An additional flat panel TV screen is powered by the third computer. The presence of additional monitors allows multiple AW windows to be visible, in addition to providing space for the separate, necessary software programs that will be needed. All software (i.e., our electronic medical record, our anesthesia information management system, and AW) either is accessible on these computers through desktop icons or links through our department’s webpage. A guide to the ACT and an AW help guide are provided in a physical format in addition to electronic copies. Two landline telephones and one iPhone will be available in the randomized control trial.

The ACT will be staffed by attending anesthesiologists and anesthesiology residents who are in their final year of training. The main role of these trained clinicians (referred to as “ACTors”) is to monitor all active ORs by making use of AlertWatch, the hospital’s EMR, and the anesthesiology information management system. ACTors will assess and address alerts within the AW Tower Mode platform (Figure 3) and will use their clinical judgment to determine whether a particular alert warrants contact with the clinicians in the OR (CORs) to provide additional support.

## Appendix 2

### Debriefing scripts

#### Debriefing script for phase Ia (ACTor sessions)

*Thank you for filling out the questionnaires. Before you leave, I would love to talk briefly about your overall impression of the ACT.*

*Right now how do you feel about the ACT after the session today?*

*Were there problems that you encountered with any of the software?*

*Were there problems that you encountered with the physical set up?*

*Was there anything in the AlertWatch program or the other software that you think worked well?*

*Was there anything in the physical set up of the ACT that you think worked well?*

#### Debriefing script for phase Ib (COR sessions)

For each case scenario:

*What thoughts come to your mind as you imagine this [scenario]?*

*Would you want to communicate with the ACT? If so, what would you communicate?*

*Do you think the ACT would be helpful in this situation?*

*If yes, what makes it helpful?*

*If not, what keeps it from being helpful?*

*Could it ever be helpful?*

For the final debriefing:

*Now that we are finished with that part [of the session], do you have additional general thoughts about the ACT?*

*What are instances in which you think it would be beneficial to receive an alert or assistance from the ACT?*

If participant cannot think of instances, prompt further: *is there any clinical situation that you can think of in which it would be helpful to receive alerts from the ACT?*

*What are instances in which it would be* ***not*** *be beneficial or helpful to receive an alert?*

*What form of alert, phone call, text, or page, would be most helpful? Would that ever change?*

*Do you have any other feedback, concerns, or questions before we end?*

## Abbreviations

ACT: Anesthesiology Control Tower
ACTor: clinician in the Anesthesiology Control Tower
AW: AlertWatch
COR: Clinician in the operating room
CRNA: Certified registered nurse anesthetist
CSUQ: Computer System Usability Questionnaire
IT: Information technology
NASA-TLX: NASA Task Loading Index
OR: Operating room
SUS: System Usability Scale
